# Simplified Dosing Regimens for Gentamicin in Neonatal Sepsis

**DOI:** 10.3389/fphar.2021.624662

**Published:** 2021-03-08

**Authors:** S. D’Agate, F. Tshinanu Musuamba, E. Jacqz-Aigrain, O. Della Pasqua

**Affiliations:** ^1^ Clinical Pharmacology and Therapeutics Group, University College London, London, United Kingdom; ^2^ Department of Paediatric Pharmacology and Pharmacogenetics, Centre Hospitalier Universitaire, Hôpital Robert Debré, Paris, France

**Keywords:** gentamicin, neonatal sepsis, pharmacokinetics, modeling and simulation, dosing optimization, bacterial infection, resource-limited and remote setting

## Abstract

**Background:** The effectiveness of antibiotics for the treatment of severe bacterial infections in newborns in resource-limited settings has been determined by empirical evidence. However, such an approach does not warrant optimal exposure to antibiotic agents, which are known to show different disposition characteristics in this population. Here we evaluate the rationale for a simplified regimen of gentamicin taking into account the effect of body size and organ maturation on pharmacokinetics. The analysis is supported by efficacy data from a series of clinical trials in this population.

**Methods:** A previously published pharmacokinetic model was used to simulate gentamicin concentration vs. time profiles in a virtual cohort of neonates. Model predictive performance was assessed by supplementary external validation procedures using therapeutic drug monitoring data collected in neonates and young infants with or without sepsis. Subsequently, clinical trial simulations were performed to characterize the exposure to intra-muscular gentamicin after a q.d. regimen. The selection of a simplified regimen was based on peak and trough drug levels during the course of treatment.

**Results:** In contrast to current World Health Organization guidelines, which recommend gentamicin doses between 5 and 7.5 mg/kg, our analysis shows that gentamicin can be used as a fixed dose regimen according to three weight-bands: 10 mg for patients with body weight <2.5 kg, 16 mg for patients with body weight between 2.5 and 4 kg, and 30 mg for those with body weight >4 kg.

**Conclusion:** The choice of the dose of an antibiotic must be supported by a strong scientific rationale, taking into account the differences in drug disposition in the target patient population. Our analysis reveals that a simplified regimen is feasible and could be used in resource-limited settings for the treatment of sepsis in neonates and young infants with sepsis aged 0–59 days.

## Introduction

Bacterial infections persist as a global health problem (UNICEF, 2018). Children mortality remains exceptionally high during the first month of life, with more than 99% of neonatal deaths occurring in developing countries. Moreover, a quarter of these deaths are attributed to neonatal sepsis (Liu et al., 2012; Chan et al., 2013; Lawn et al., 2014). The recommended initial empirical therapy for a neonate with suspected bacterial sepsis and/or meningitis includes ampicillin and an aminoglycoside (Zaidi et al., 2011; Polin et al., 2012; World Health Organization, 2015; Seale et al., 2015). This combination expands the antimicrobial spectrum and can be prescribed at a considerably low cost (Lee et al., 2014). However, despite the availability of clinical guidelines and recommendations for the treatment of serious bacterial infections in resource-limited settings, where the recommended inpatient treatment may not be feasible, challenges still exist in the effective delivery of life-saving drugs to this vulnerable population (Lassi et al., 2010; Esamai et al., 2013; African Neonatal Sepsis Trial et al., 2015; Simen-Kapeu et al., 2015). In addition to accessibility, acceptability or affordability issues, this is also due to the complexity of the recommended dosing regimens, which have been introduced into clinical practice in a rather empirical manner. Such an approach does not warrant optimal exposure of newborns to antibiotic agents, which show a different disposition profile in this population (Cella et al., 2010a; Medellin-Garibay et al., 2015; Samardzic et al., 2016).

In fact, a few historical antibiotic efficacy trials were performed in neonates >20 years ago and these have been conducted without careful evaluation of the implications that differences in drug disposition represent for the dose rationale (Evans et al., 1986; Buring et al., 1988; Wiese, 1988; Fisk, 1993). By contrast, a vast body of evidence is currently available that allows one to assess the role of age and disease-related changes in drug disposition and overall differences in the pharmacokinetic properties of antibiotics (Manolis and Pons, 2009; Pandolfini et al., 2013; Roberts et al., 2014; Stockmann et al., 2014; Pineda and Watt, 2015). Here we assess the feasibility of a simplified regimen of gentamicin taking into account the effect of body size and organ maturation on pharmacokinetics. Using quantitative clinical pharmacology methods, and more specifically clinical trial simulations, we characterize the impact of covariate factors on the disposition of gentamicin in preterm and term infants aged 0–59 days. The aim of this analysis was to evaluate the performance of it is possible to evaluate current World Health Organisation (WHO) guidelines, which recommend gentamicin doses between 5 and 7.5 mg/kg and identify a simplified regimen for the use of intramuscular gentamicin in resource-limited settings.

Making use of clinical trial simulations, it is possible to evaluate clinically relevant scenarios including the effect of intrinsic (e.g., disease) and extrinsic (e.g., co-medication) factors known to alter the pharmacokinetic properties of a drug (Anderson, 2010; Bellanti and Della Pasqua, 2011). These considerations are especially important in very young pediatric patients, who are not simply small in terms of total body size or surface area. They differ in terms of organ function capacity, ontogeny and maturation, all of which can affect pharmacokinetic processes in a nonlinear manner, and consequently lead to differences in systemic and target tissue exposure (Cella et al., 2010b; Rodieux et al., 2015). Such a nonlinearity implies that the use of dosing recommendations on a milligram per kilogram basis (mg/kg) does not necessarily correct for the underlying differences in pharmacokinetics.

Yet, efficacy findings supporting dose recommendations have often overlooked the importance of exposure and pharmacokinetic-pharmacodynamic data (Zaidi et al., 2013a; Baqui et al., 2015). In this regard, it is important to underline that the antibacterial activity of gentamicin is concentration-dependent, as expressed by the ratio of peak plasma concentration over the minimum inhibitory concentration (C_max_: MIC), which should exceed 8–10 for optimal efficacy (Moore et al., 1987; Levison and Levison, 2009). Nevertheless, attainment and maintenance of target levels may be challenging in preterm and term newborn infants, as changes in organ function occur relatively fast. Gentamicin is essentially eliminated by renal excretion through glomerular filtration and as such its elimination is determined by nephrogenesis, which reaches completion between 32 and 36 weeks of gestation (Rodriguez et al., 2004; Schreuder et al., 2011). In addition, gentamicin disposition is affected by distributional differences, such as extracellular body water and changes in renal blood flow. During the first weeks of life, there is a progressive rise in glomerular filtration rate (GFR) resulting from an acute increase in cardiac output induced by a decrease in renal vascular resistance (Vanpee et al., 1992; Andersen et al., 2012). Consequently, renal elimination of gentamicin in neonates is largely linked to both gestational age and post-natal (PNA) age.

The selection of a dose and dosing regimen for the neonatal population should therefore account for the effect of maturation (increase in age and function) and growth (increase in size). Ultimately, we envisage the possibility of deriving simplified fixed dose regimens for intramuscular gentamicin, which will facilitate prescription and dispensation practice in a resource-limited setting whilst minimizing the risk of under and overexposure of preterm and term neonates to antibiotics.

## Materials and Methods

### Clinical Data

Demographic and clinical response data from patients who were enrolled into the AFRINEST and SATT trials were used as reference for the purpose of the current investigation. The availability of these data allows for resampling of relevant covariates taking into account actual distributions and correlations between demographic and clinical baseline characteristics. As shown in Supplementary Figure S1, data for post-natal age presents a skewed distribution, which adequately reflects the epidemiologic characteristics of sepsis after birth. An overview of the demographic variables included in the analysis is presented in Table 1. Further details of the trial protocols are available elsewhere (Zaidi et al., 2013b; African Neonatal Sepsis Trial et al., 2015; Baqui et al., 2015). The studies have been conducted in full conformance with the principles of the Declaration of Helsinki and with the local laws and regulations concerning clinical trials.

**TABLE 1:** Demographic characteristics of the pediatric patients enrolled in the AFRINEST and SATT trials (Zaidi et al., 2013b; African Neonatal Sepsis Trial et al., 2015; Baqui et al., 2015), which were used in subsequent simulation scenarios.

Patient characteristics	Value
Number of patients	10,840
Post-natal age (days), median (range)	16 (1–59)
Weight (kg), median (range)	3.3 (1.5–8)
Male, %	51

### Virtual Population Cohort

The baseline data were used to create a virtual cohort with similar characteristics of the pediatric patients enrolled in the AFRINEST and SATT trials. Of interest were the demographic characteristics which have been identified as influential covariates on disposition parameters. As information on gestational age (GA) was not available for individual patients in the clinical trials, GA was imputed from post-menstrual age (PMA), body weight (BW) and gender using the approach described by Sumpter and Holford (Sumpter and Holford, 2011). The method relies on the assumption of a correlation between actual body weight, weight at birth and gestational/post-natal age. To prevent spurious variability in subsequent simulation steps and avoid combinations of individual BW and GA which might not be biologically plausible, each individual patient was assigned a GA value that corresponded to the median of the predicted GA distribution for the patient’s body weight. Subsequently, the proportion of preterm infants with imputed GA between 24 and 36 weeks was compared with epidemiological data describing the prevalence of preterm births (Tucker and McGuire, 2004; Chawanpaiboon et al., 2019), which occur on average in 12% of the population. In addition, predicted values were further compared against data describing the incidence of sepsis between birth and 59 days (Waters et al., 2011; Blencowe et al., 2013; Downie et al., 2013). An overview of the correlations between body weight, GA and PNA for preterm and term newborns and infants is shown in Supplementary Figure S2.

### Pharmacokinetic Model Selection

The search strategy for model selection included the following keywords and MESH terms in PubMed: “gentamicin,” “pharmacokinetics,” “model,” “sepsis,” “neonates” and/or “infants.” Three important criteria were used for inclusion of publications, namely, 1) Comparable demographics to the population in our analysis, 2) No confounding comorbidity or comedications and 3) Internal and external validation procedures. An additional exclusion criteria regarded the choice of parameterization used to describe the disposition properties of gentamicin. Models based on empirical parameterization were deemed unsuitable for prospective simulations. Initially, five candidate models were identified that seemed relevant for the purposes of the current investigation (Supplementary Table S1). However, after careful review of the publications only the model proposed by Fuchs and colleagues appeared to satisfy all the predefined inclusion and exclusion criteria (Fuchs et al., 2014). Moreover, the model was developed using a very large population including newborns and infants from 24 to 42 weeks gestational age (namely, 994 preterm and 455 term newborns). Such a large population ensured parameter estimates with higher precision, as well as accurate characterisation of the interindividual variability in disposition characteristics in this group of patients.

**FIGURE 1:**
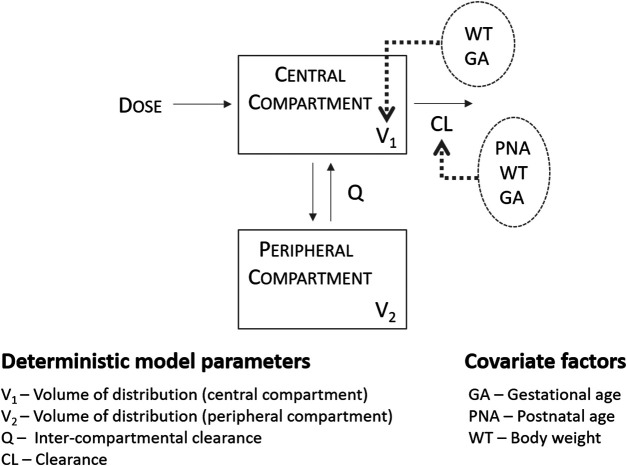
Population pharmacokinetics of gentamicin in a large cohort of preterm and term neonates, as described by Fuchs et al. (2014) Absorption was assumed to be instantaneous and suitable for the description of gentamicin profiles following intramuscular administration.

In brief, Fuchs and colleagues have identified a two-compartment pharmacokinetic model to describe the disposition of gentamicin in preterm and term neonatal patients following intravenous administration over a 30 min infusion, most of the time in association with amoxicillin. Model evaluation was based on standard graphical and statistical criteria and included external validation procedures. The average parameter estimates and corresponding between-subject variability (BSV%) for a median body weight of 2.17 kg were 0.089 L/h (28%) for clearance (CL) and 0.908 L (18%) for the central volume of distribution (Vc). Body weight, gestational age and post-natal age positively influenced CL, whereas body weight and gestational age positively influenced the volume of distribution of the central compartment (Figure 1; Supplementary Table S2 for details on the pharmacokinetic parameter estimates). To ensure that model parameters and covariate-parameter correlations were coded correctly, an initial evaluation was performed to assess model performance. The model by Fuchs et al. was used to simulate gentamicin plasma concentrations in sepsis patients described in Thomson et al. (Thomson et al., 2003).

Subsequently, the generalisability of the pharmacokinetic model for the simulation of gentamicin concentration vs. time profiles was assessed in a population that reflects real-world conditions. An additional evaluation, from now on referred to as secondary external validation was performed using therapeutic drug monitoring data collected in subjects with or without sepsis at the Robert Debré Children’s Hospital, Paris, as part of therapeutic drug monitoring. Initially, 37 plasma samples from 29 subjects with comparable demographic baseline characteristics, who were treated with standard gentamicin intravenous doses were retrieved for the secondary external validation. Of these, one subject was excluded from the data set because of inaccurate details on the reported dosing regimen. An overview of the demographic variables of the subjects used for model building in Fuchs et al. and for the secondary external validation is presented in Table 2. For the evaluation of model performance, *post-hoc* parameter estimates were obtained for each subject in the secondary external validation data set by using the MAXEVAL = 0 option. Then, goodness-of-fit (GOF), visual predictive checks (VPC) and model prediction error (MPE) were assessed. The VPC was generated using 1,000 simulations. Median and 90% confidence intervals of the simulated values were calculated for each subject and plotted together with the observed data. The mean percentage error (MPE) was calculated according to the following formula:

$$MPE\;\left(\%\right)=\frac{IPRED-DV}{DV}\cdot 100$$

where IPRED is the individual predicted concentration and DV is the observed concentration.

**TABLE 2:** Demographic characteristics of patients used for model building and secondary external validation.

Patient characteristics	Model building *	Secondary external validation
Number of patients	1,449	28
Gestational age (weeks), median (range)	34 (24–42)	37 (24.7–41)
Post-natal age (days), median (range)	1 (0–94)	4.5 (1–145)
Post-menstrual age (weeks), median (range)	34.4 (24.2–42.4)	37 (25.9–60)
Weight (kg), median (range)	2.2 (0.4–5.5)	2.9 (0.6–4.5)
Male, %	57.5	54

* Values from model building are adapted from Fuchs et al. (2014) with permission.

In addition, the positive predicted value (PPV) and the negative predicted value (NPV) were calculated to evaluate the ability of the model to correctly predict trough concentrations below the safety threshold (2 mg/L). Finally, the secondary parameters of interest (AUC, C_max_ and C_min_) were derived from the predicted pharmacokinetic profiles using noncompartmental methods.

### Simulation Assumptions

As the current evaluation is part of a broader investigation aimed to identify the feasibility of simplified regimens for first line antibiotics for the treatment of severe bacterial infections when referral is not possible, a common set of assumptions has been used for each of the drugs in scope. As presented previously by D’Agate and colleagues for amoxicillin (D’Agate et al., 2020), six key assumptions were required for the assessment and interpretation of the results, namely:

1 Treatment failure was assumed to be linked to pharmacokinetic variability (i.e., underexposure), rather than resistance (Roberts and Lipman, 2006).
2 Correlations between patient demographic characteristics and physiological processes that determine interindividual differences in drug disposition were considered to be constant across the course of disease, unless stated otherwise.
3 The absorption rate of gentamicin after intramuscular administration was deemed to be very fast and as such for modeling purposes, dose was assumed to be delivered directly into the central compartment, as per parenteral administration. This assumption is supported by previous investigations, which have shown comparable disposition profiles of gentamicin after intramuscular and intravenous administration (Hayani et al., 1997; Gemer et al., 2001). In addition, Seaton et al. and Thomson et al. both showed that a first order absorption model does not describe intramuscular pharmacokinetic data in infants and children (Thomson et al., 2003; Seaton et al., 2007).
4 Dose proportionality (i.e., pharmacokinetic linearity) was assumed beyond the observed range of doses and concentrations if higher doses (i.e., up to two-fold) were used in simulation scenarios.
5 Differences of up to 10% in median secondary pharmacokinetic parameter estimates (AUC, C_max_, and C_min_) were not deemed clinically relevant. Such a variation allows for the effect of model uncertainty whilst taking into account the impact of changes in dosage forms, as defined by current regulatory guidelines, which permit even larger variation when evaluating whether different formulations are bioequivalent.
6 Treatment adherence was assumed to vary randomly and to be dose-independent for the purposes of simulations.

### Simulation Scenarios—*In Silico* Clinical Trial Protocol

**FIGURE 2:**
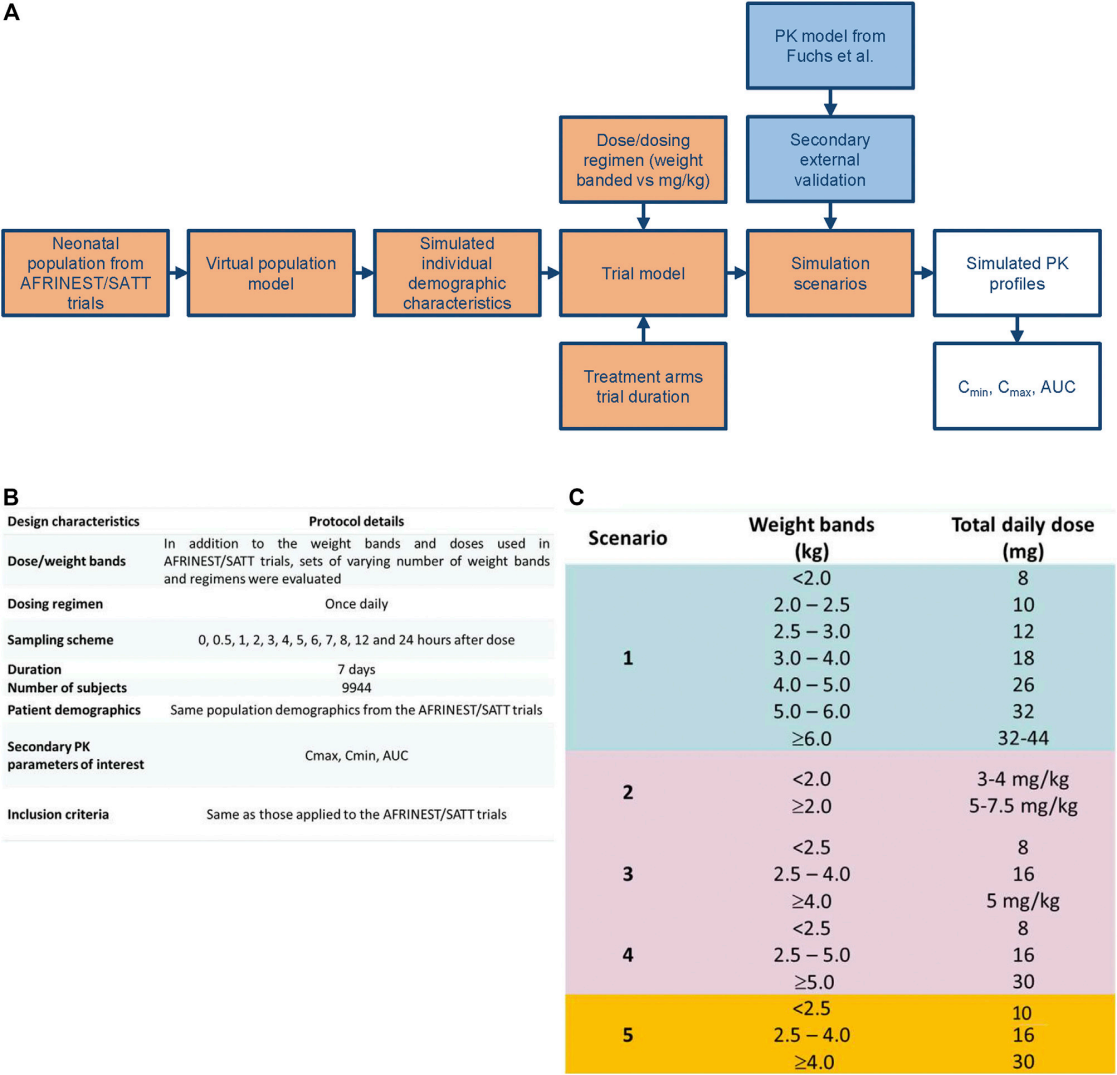
**(A)** Workflow for the implementation of clinical trial simulations aimed at the evaluation of simplified dosing regimens for gentamicin in neonatal sepsis. **(B)** Design characteristics for the study protocol used in the clinical trial simulations. **(C)** Simulation scenarios evaluated for the identification of a simplified dosing regimen based on weight bands and fixed doses.

Gentamicin exposure following once daily intramuscular administration was simulated in a virtual cohort (*n* = 9,994) of preterm and term newborns and infants with post-natal age varying from 0 to 59 days. In addition to the effect of demographic covariates simulation scenarios have also accounted for the skewed distributions in age and body weight, which reflects the incidence and prevalence of sepsis in this population. An outline of the simulation steps and selected scenarios for the identification of a simplified regimen are summarized in Figure 2. The currently recommended dosing regimen by WHO was used as reference scenario for the purpose of comparisons between regimens (World Health Organization, 2015).

The parameters of interest included the peak concentration (C_max_) and the trough concentration (C_min_) associated with once daily gentamicin administration. In addition, the area under the curve (AUC) was calculated to compare exposure across weight and age groups in the population as this parameter correlates with the total dose delivered, which is currently given in mg/kg. Given that gentamicin is delivered intramuscularly and absorption is rapid, formulation was not considered a significant source of variability in the simulation scenarios (Thomson et al., 2003).

All simulation scenarios were based on the use of once daily doses of gentamicin for a period of 7 days. Pharmacokinetic sampling was implemented according to a typical sampling scheme with one pre-dose and 11 post-dose samples (Figure 2). Even though optimization techniques have not been applied, the selected sampling intervals were assumed to allow accurate estimation of AUC over the dosing interval as well as identification of peak and through levels of gentamicin. C_max_ and C_min_ were calculated, respectively, by taking the maximum predicted concentration and the predicted pre-dose concentration after the first dose and at steady state conditions at the end of treatment. For the sake of comparison, integration of the concentration vs. time data according to the trapezoidal rule was applied for the calculation of the AUC. All simulations were performed using NONMEM version 7.3 (ICON Development Solutions, Ellicott City, MD, United States). R version 3.1.2 (R Core Team, 2013) was used to graphically summarize the results.

Threshold values for target peak and trough concentrations were selected for comparison of the performance of the different dosing regimens taking into account pharmacokinetic-pharmacodynamic indices as well as microbiological susceptibility data, safety and efficacy results from available clinical studies. A cut-off value of 10 mg/L was used for peak concentrations and 2 mg/L for trough concentrations. These thresholds were used as a proxy for efficacy and safety, respectively. Consequently, the selected regimens aimed at maximizing the proportion (in percentage, %) of sepsis patients aged 0–59 days with peak concentrations above the reference threshold level of 10 mg/L, whilst minimizing those below the 2 mg/L threshold for trough concentrations. The reference thresholds were based on recommendations from the British National Formulary (https://bnf.nice.org.uk/drug/genta-micin.html) and a comprehensive review on the use of gentamicin for the treatment of suspected or proven sepsis (Rao et al., 2015; Ibrahim et al., 2016). Given the aforementioned criteria, no formal hypothesis test was used to compare scenarios. Each scenario was summarized taking into account the weight bands associated with the corresponding scenario. Median estimates were calculated along with the 90% confidence intervals for the parameters of interest.

## Results

Our analysis shows how doses and dosing regimens can be evaluated in a systematic manner, considering the contribution of factors known to affect drug disposition in the neonatal patient population. In addition to the selected simplified regimen, two scenarios are discussed: 1) the performance of the dosing regimens used in AFRINEST and SATT studies and 2) the 2015 WHO recommendations for management of possible serious bacterial infections in young infants 0–59 days old when referral is not feasible (World Health Organization, 2015). Predicted concentration vs. time profiles, peak and through concentrations in the AFRINEST and SATT studies were used as basis for further assessment and interpretation of the role of interindividual variability in drug disposition in the neonatal population.

### Model Performance and Secondary External Validation

The secondary external validation procedures showed that model predictions for trough concentrations are associated with a median MPE of −7.7%. This was slightly higher than the median MPE reported for the external validation in Fuchs et al. (i.e., −3%). Clearly, the higher variability observed for the MPE in this group of patients reflects the heterogeneity of the pediatric population. Yet, the model showed adequate performance, predicting correctly whether a trough concentration is below the threshold for safety (2 mg/L) with a PPV of 0.94 and an NPV of 0.87.

The goodness-of-fit plots for the secondary external validation data set are shown in Supplementary Figure S3, together with the data from the external validation performed by Fuchs et al. These plots indicate comparable model performance for the different data sets, but with a higher unexplained variability. An overview of the concentration vs. time profiles is depicted in Supplementary Figure S4, where the individual VPCs show the predictive performance of the model, especially for lower concentrations. The predicted median AUC was 110 mg h/L (90% CI 49–129 mg h/L), C_max_ was 14.4 mg/L (90% CI 10.6–20.9 mg/L) and C_min_ was 0.66 (90% CI 0.07–2.58 mg/L).

### Predicted Gentamicin Exposure in the AFRINEST and SATT Studies

**FIGURE 3:**
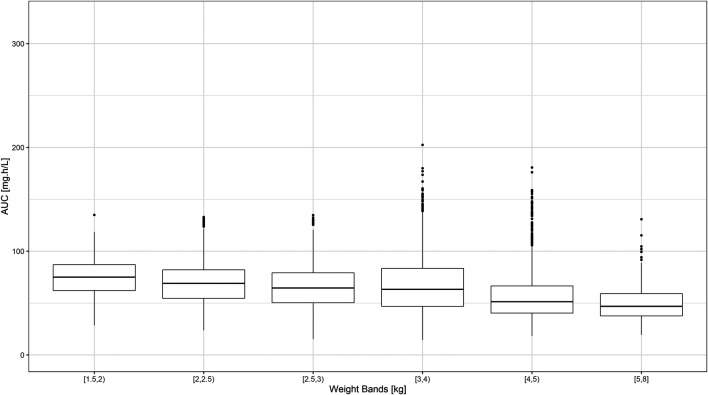
Predicted gentamicin AUC in sepsis patients aged between 0–59 days. Estimates are summarized according to the weight bands used in the original AFRINEST/SATT trials. Hinges represent 25^th^ and 75^th^ percentiles (respectively, Q1 and Q3), whiskers represent Q1 − 1.5IQR and Q3 + 1.5IQR, respectively, where IQR is the interquartile range. All the subjects outside this range are represented by the dots (*N* = 9,994).

Despite the use of six weight bands to account for the effect of body weight, the median exposure to gentamicin, expressed as the area under concentration vs. time curve, was found to vary by more than 50% across the different groups (Figure 3). Whilst most subjects appear to achieve target peak and trough concentrations of gentamicin, considerable fluctuation in drug levels was observed across the different weight bands. Target concentrations are not achieved in a small proportion of subjects in the lower weight bands.

**FIGURE 4:**
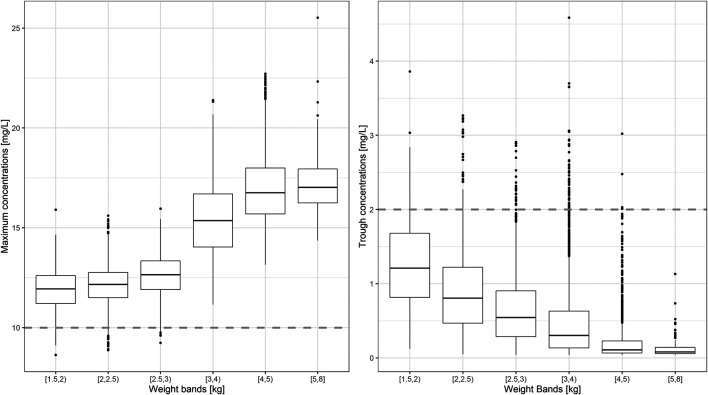
Predicted gentamicin peak **(left)** and trough **(right)** concentrations in sepsis patients aged between 0 and 59 days. Estimates are summarized according to the weight-bands used in the original AFRINEST/SATT trials. Hinges represent 25^th^ and 75^th^ percentiles (respectively, Q1 and Q3), whiskers represent Q1 − 1.5IQR and Q3 + 1.5IQR, respectively, where IQR is the interquartile range. All the subjects outside this range are represented by the dots (*N* = 9,994). Dashed lines represent the threshold values for peak and trough concentrations of 10 and 2 mg/L, respectively.

An overview of the variability in the predicted peak and trough concentrations of gentamicin is shown in Figure 4, where C_max_ and C_min_ values are summarized after the first dose of a once daily dosing regimen over a period of 7 days. Data were stratified by weight bands, as per protocol. In addition, the predicted percentage (%) of sepsis patients with peak concentrations below the reference threshold level of 10 mg/L and trough concentrations above 2 mg/L is summarized in Table 3.

**TABLE 3:** Predicted percentage (%) of sepsis patients aged 0–59 days with peak concentrations below the reference threshold level of 10 mg/L and trough concentrations above 2 mg/L after a once daily dosing regimen of gentamicin.

Trial weight band (kg)	No. patients/weight band	% of patients with C_max_ < 10 mg/L				No. of patients with C_max_ < 10 mg/L			
		AFRINEST/SATT study regimen	Proposed regimen	WHO (lower doses^a^)	WHO (higher doses^b^)	AFRINEST/SATT study regimen	Proposed regimen	WHO (lower doses^a^)	WHO(higher doses^b^)
1.5–2.0	294	3.7	0.0	98.3	17.7	11	11	289	52
2.0–2.5	820	4.1	4.1	12.2	0.0	34	34	100	0
2.5–3.0	1,783	1.1	0.0	0.0	0.0	20	0	0	0
3.0–4.0	4,656	0.0	0.1	0.0	0.0	0	3	0	0
4.0–5.0	2,081	0.0	0.0	0.0	0.0	0	0	0	0
5.0–8.0	360	0.0	0.0	0.0	0.0	0	0	0	0

Trial weight band (kg)	No. patients/weight band	% of patients with C_min_ > 2 mg/L				No. of patients with C_min_ > 2 mg/L			
		AFRINEST/SATT study regimen	Proposed regimen	WHO (lower doses^a^)	WHO (higher doses^b^)	AFRINEST/SATT study regimen	Proposed regimen	WHO (lower doses^a^)	WHO (higher doses^b^)
1.5–2.0	294	12.6	27.6	0.3	6.1	37	81	1	18
2.0–2.5	820	5.1	5.1	6.0	26.2	42	42	49	215
2.5–3.0	1,783	1.4	6.6	2.5	13.6	25	117	45	242
3.0–4.0	4,656	1.3	0.7	0.7	4.0	59	34	31	185
4.0–5.0	2,081	0.2	0.4	0.0	0.6	4	9	1	12
5.0–8.0	360	0.0	0.0	0.0	0.0	0	0	0	0

^a^ Lower doses are 3 mg/kg for low birth weight (<2.0 kg) newborns and 5 mg/kg for those with body weight >2.0 kg.

^b^ Higher doses are 4 mg/kg for low birth weight newborns and 7.5 mg/kg for those with body weight >2.0 kg.

Data are summarized according to the weight bands used in the trials.

### Comparison Between the Proposed Simplified Regimen and the WHO Recommendations

**FIGURE 5:**
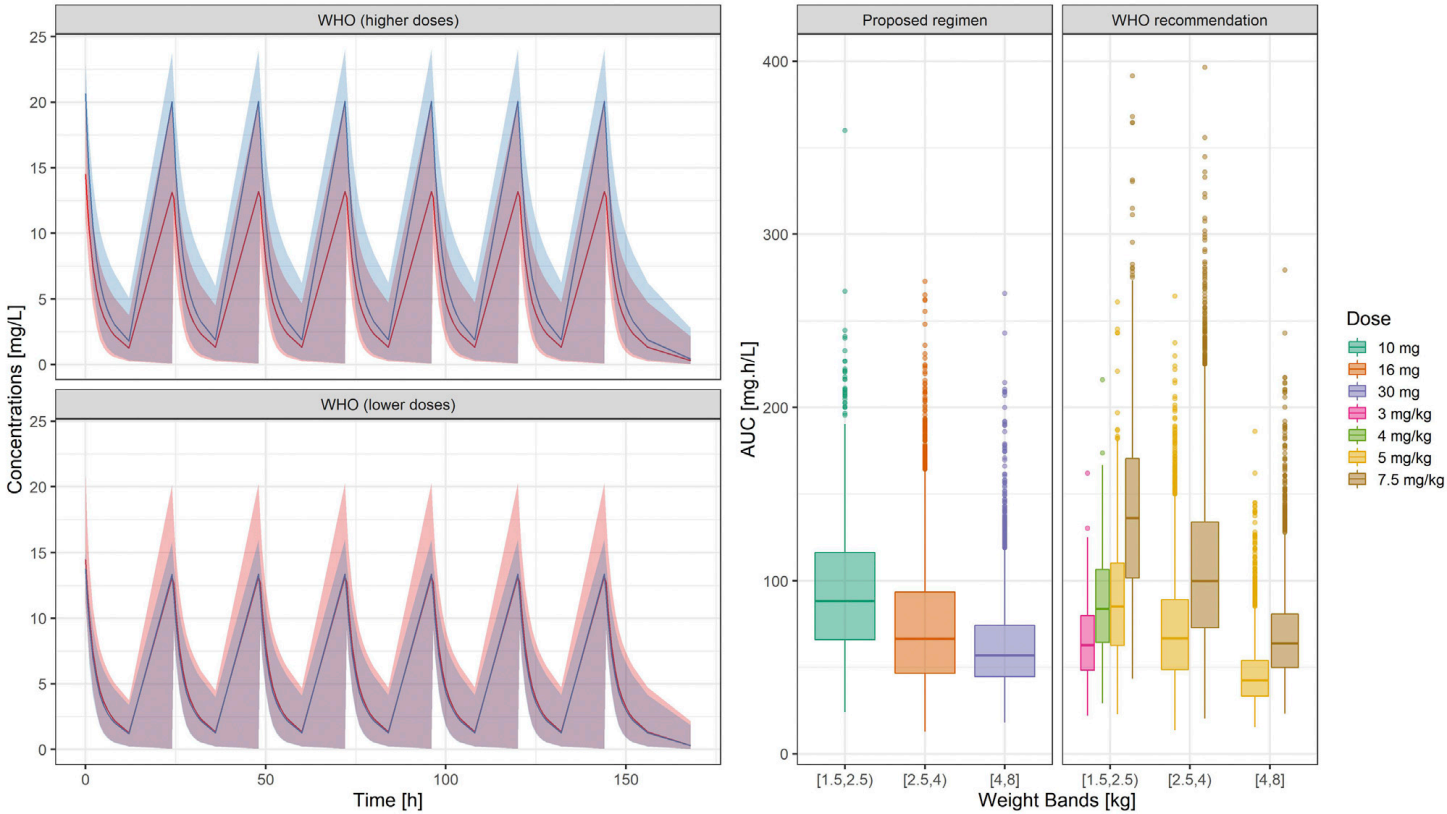
**(Left)** Predicted gentamicin concentration vs. time profile in sepsis patients aged between 0 and 59 days. Each panel compares the pharmacokinetic profiles in the target population after the proposed regimen (red) with those obtained after higher (upper panel) and lower doses (lower panel) of the WHO recommended regimen (blue). Solid line depicts the median profile; shaded area represents the 90% prediction intervals. Time 0 is used as reference for the first dose **(Right)** Systemic exposure, expressed as area under the concentration vs. time curve. Hinges represent 25^th^ and 75^th^ percentiles (respectively, Q1 and Q3), whiskers represent Q1 − 1.5IQR and Q3 + 1.5IQR, respectively, where IQR is the interquartile range. All the subjects outside this range are represented by the dots (*N* = 9,994). Overall, the weight-banded regimens result in similar exposure ranges, with a slight trend to lower values in the highest weight band.

In contrast to current guidelines, which recommend the use of gentamicin in mg/kg, our analysis demonstrates the feasibility of implementing a fixed dose regimen based on three weight bands. Figure 5 shows the population predicted plasma concentration vs. time profile of gentamicin for the proposed simplified regimen along with the 90% confidence intervals, as compared to the 2015 WHO recommended doses of 5.0 – 7.5 mg/kg. As it can be observed, the two regimens seem to overlap considerably with each other.

**FIGURE 6:**
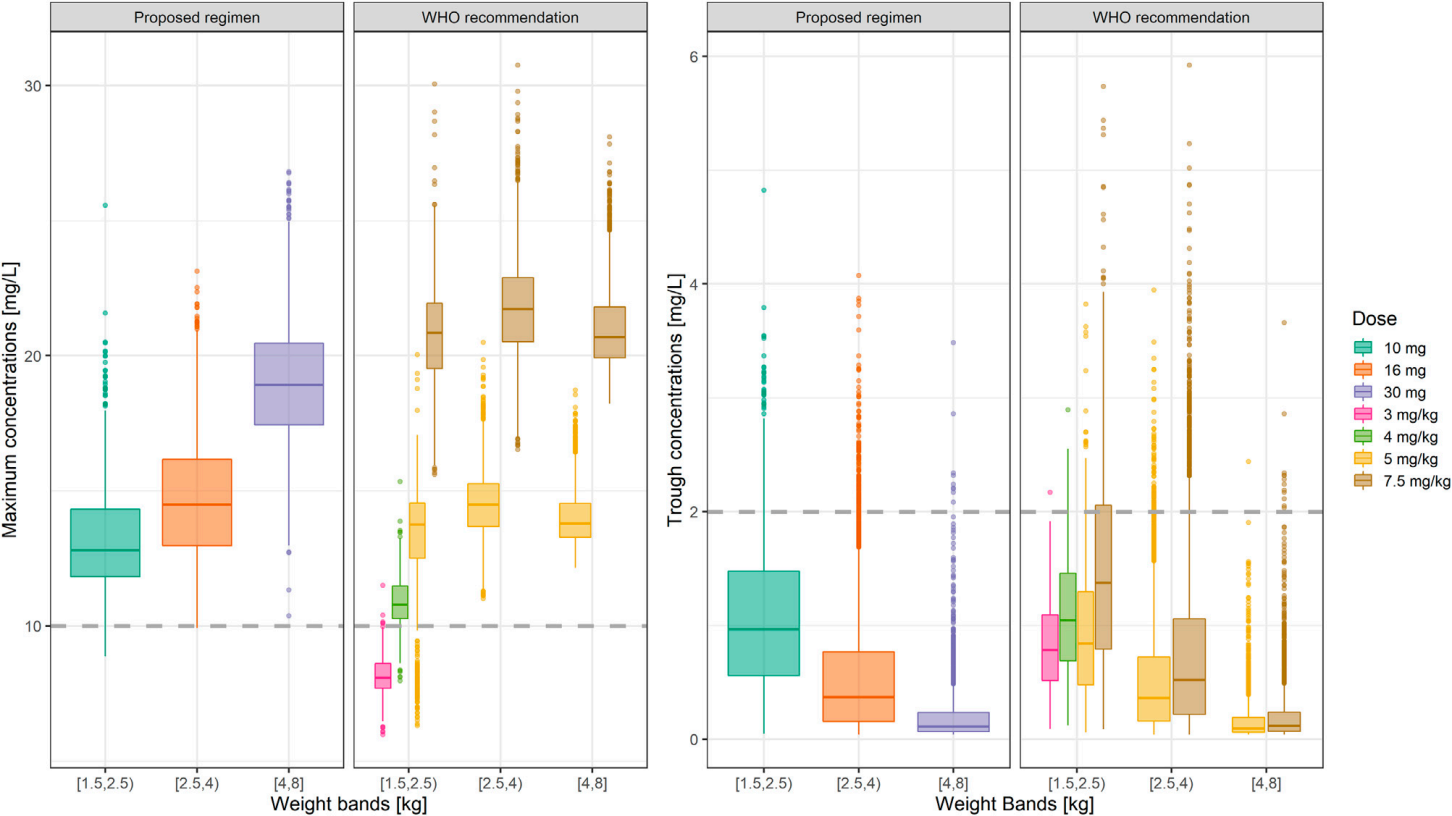
Predicted gentamicin peak **(left)** and trough **(right)** concentrations in sepsis patients aged between 0 and 59 days stratified according to the weight bands for the proposed simplified regimen. Panels show how the simplified regimen compares to the 2015 WHO recommendations. Hinges represent 25^th^ and 75^th^ percentiles (respectively, Q1 and Q3), whiskers represent Q1 − 1.5IQR and Q3 + 1.5IQR, respectively, where IQR is the interquartile range. All the subjects outside this range are represented by the dots (*N* = 9,994). Dashed lines represent the threshold values for peak and trough concentrations of 10 and 2 mg/L, respectively.

Summary statistics of the two main secondary parameters (C_max_ and C_min_) are presented along with the 90% confidence intervals in Table 4. An overview of the variability in the predicted peak and trough concentrations of gentamicin is shown in Figure 6, where data are stratified by weight bands. It is clear from the results that despite correction for differences in body weight, considerable variation is observed between lower and upper weight ranges. Given the possibility of selecting gentamicin doses between 5.0 and 7.5 mg/kg, some children remain significantly below the target threshold for peak concentrations, whilst others exceed the threshold of 2 mg/L. As can be seen from Table 3, the proposed simplified regimen represents an opportunity for dose optimization not only with respect to the current WHO recommended doses, but also when considering more complex regimens, as those implemented in the AFRINEST/SATT trials.

**TABLE 4:** Predicted peak (C_max_) and trough (C_min_) concentrations of gentamicin after a once daily dosing regimen.

Weight band (kg)	C_max_ (mg/L)				C_min_ (mg/L)			
	AFRINEST/SATT study regimens	Proposed regimen	WHO (lower doses^a^)	WHO (higher doses^b^)	AFRINEST/SATT study regimens	Proposed regimen	WHO (lower doses^a^)	WHO (higher doses^b^)
1.5–2.5	12.37 (10.2, 14.88)	12.8 (10.46, 17.33)	12.(7.35, 15.69)	20 (10.06, 23.6)	0.9 (0.2, 2.15)	0.96 (0.2, 2.45)	0.82 (0.19, 1.93)	1.24 (0.3, 3.04)
2.5–4.0	14.55 (11.58, 18.28)	14.49 (11.27, 18.47)	14.48 (12.44, 16.34)	21.73 (18.66, 24.51)	0.37 (0.06, 1.48)	0.37 (0.06, 1.64)	0.36 (0.06, 1.47)	0.52 (0.08, 2.19)
4.0–8.0	16.82 (14.48, 20.07)	18.(15.38, 23.1)	13.79 (12.7, 16.02)	20.69 (19.05, 24.03)	0.1 (0.04, 0.67)	0.11(0.04, 0.75)	0.09 (0.04, 0.57)	0.12 (0.05, 0.79)

Values shown are the median, 5^th^ and 95^th^ percentiles.

^a^ Lower doses are 3 mg/kg for low birth weight (<2.0 kg) newborns and 5 mg/kg for those with body weight >2.0 kg.

^b^ Higher doses are 4 mg/kg for low birth weight newborns and 7.5 mg/kg for those with body weight >2.0 kg.

Of note are the changes in disposition characteristics in pre-term low weight newborns and infants, especially for subjects between 1.5 and 2.0 kg, as doses of 3.0 mg/kg lead to a significant proportion of subjects below the target peak concentration. The proposed regimen practically eliminates the problem, with all subjects reaching C_max_ values greater than 10 mg/L. However, an increase in the proportion of subjects with C_max_ > 2 mg/L is also observed. The 95% percentile of C_min_ in this subgroup of subjects is 2.45 mg/L.

In addition, to assess the implications of the different regimens, data were also presented using narrower weight bands (Supplementary Figure S5). Our results reveal that a considerable number of patients <2 kg appear to remain below the target threshold for peak concentrations following a 3.0 mg/kg dose. This situation is corrected by the proposed simplified regimen. Whereas the difference between the proposed regimen and WHO recommendations are small, heterogeneity in renal maturation may drive the variation observed in trough levels in newborns with body weight between 2.0 and 2.5 kg. The simplified regimen presented in Table 5 is therefore preferable and should be used as final recommendation for the treatment of neonatal sepsis.

**TABLE 5:** Proposed simplified regimen based on fixed doses of gentamicin for the treatment of sepsis patients aged between 0 and 59 days.

Weight band	Body weight range (kg)	Simplified regimen (mg)	Volume of gentamicin 40 mg/ml administered per dose (ml)
1	1.5–2.5	10	0.25
2	>2.5–4.0	16	0.40
3	>4.0–8.0	30	0.75

## Discussion and Conclusion

Currently, the WHO recommends the use of gentamicin in combination with ampicillin or amoxicillin as empirical therapy for sepsis in newborns and infants (0–59 days old) (World Health Organization, 2015). Recent clinical trials in this vulnerable population, such as AFRINEST and SATT have shown promising findings, in that high efficacy rates have been achieved with a dosing regimen that can be implemented in community-based settings (Zaidi et al., 2013a; Zaidi et al., 2013b; African Neonatal Sepsis Trial et al., 2015; Baqui et al., 2015). However, the dosing regimens used in these trials remain complex and as such do not warrant compliance in clinical practice (African Neonatal Sepsis Trial Group, 2013). Consequently, response to treatment may not be comparable to that observed during the trials. In fact, the use of mg/kg may represent an important hurdle for the implementation of such interventions in a community-based setting. Here we have shown how increasing understanding of the pharmacokinetics and pharmacokinetic-pharmacodynamic (PKPD) relationships of the antibiotics can be used in conjunction with quantitative clinical pharmacology principles to guide the dose rationale for gentamicin in newborns and infants with sepsis (Vinks et al., 2015; Mehrotra et al., 2016).

Any attempt to optimize dose and simplify dosing regimens will require therefore an alternative, less empirical approach than clinical evidence of efficacy (Khan and Joseph, 2012; Rao et al., 2015). Given that gentamicin exhibits concentration-dependent bactericidal activity and prolonged post-antibiotic effects, it is essential to understand how drug levels vary across different subgroups in the target patient population, such as infants and newborns. Ultimately, it appears that it is the amount of drug (e.g., C_max_ relative to the MIC) rather than the dosing frequency that determines the treatment response (van Maarseveen et al., 2016). Therefore, in our analysis, we have used a target threshold for C_max_ of 10 mg/L, rather than a range of concentrations or the C_max_/MIC ratio. This decision allowed for direct assessment of the observed drug levels and potential implications for the overall efficacy and safety profile of gentamicin. Furthermore, the use of this criterion implies that exposure levels can be considered efficacious for susceptible pathogens with MIC values lower or equal to 1 mg/L.

Historically, the dosing regimens used for gentamicin have evolved from multiple daily dosing to extended-interval dosing both in adults and in children (Kent et al., 2014). These regimens have aimed at ensuring that peak blood concentrations are sufficiently high to elicit a therapeutic response while avoiding high trough concentrations, which could be potentially toxic after prolonged treatment (Hoff et al., 2009; Dersch-Mills et al., 2012; Radivoyevitch et al., 2015). However, pharmacokinetic data in infants exhibits large inter- and intra-individual variability, mainly because of the developmental changes occurring from the first month of life. As a consequence, gentamicin dosing regimens based on mg/kg body weight may not fully correct for age-related changes in organ function, composition, maturation and growth (Koren et al., 1985; Salgado et al., 2010; Rodieux et al., 2015).

In contrast to most of the published pharmacokinetic and PKPD data available in the scientific literature (Lingvall et al., 2005; Nielsen et al., 2009; Sherwin et al., 2009; Roberts et al., 2010; Mohamed et al., 2012; Chin et al., 2013; De Cock et al., 2014; Sampson et al., 2014; Valitalo et al., 2015), our investigation has not been limited to a small group of patients. In fact, we have been able to evaluate pharmacokinetic variability across a large patient population, including the impact of demographic baseline covariates and other relevant disease-related factors on the disposition of gentamicin in neonates and infants. Our analysis has included a range of scenarios aimed at demonstrating the feasibility of a simplified dosing regimen with gentamicin, which accounted for relevant sources of variability in pharmacokinetics. Of note is the identification of weight bands that can be used in combination with fixed dose levels, while ensuring acceptable target peak and trough concentrations of gentamicin. Indeed, a simplified regimen that minimizes the proportion of patients below the peak concentration threshold of 10 mg/L, whilst maximizing the proportion of patients below the trough concentration threshold of 2 mg/L was identified based on the use of three weight bands, namely, <2.5, 2.5–4.0, and >4.0 kg. These cut-off values were selected taking into account the weight categories currently used for the other antibiotics indicated for the treatment of sepsis when referral is not possible (Baqui et al., 2015; World Health Organization, 2015). It should be noted that the predicted differences in exposure between the proposed simplified regimen and WHO recommendations are unlikely to be clinically relevant, with exception of patients weighting <2.5 kg. Our simulations reveal that peak concentrations will be significantly lower in this weight band after the use of gentamicin doses based on the WHO guidelines. Therefore, a dose of 10 mg should be considered for this group of patients, even if this regimen may be associated with trough levels that are slightly above 2 mg/L (95% CI: 0.2–2.45).

From a clinical perspective, in addition to demonstrating the feasibility of an alternative regimen for effective treatment of sepsis, our work also illustrates the role of comprehensive clinical trial simulations for the optimization of therapeutic interventions. We have shown how virtual patient cohorts can be created for the evaluation of interindividual differences in pharmacokinetic disposition taking into account the effect of demographic, physiological and clinical factors known to alter the distribution and elimination of gentamicin in newborns and infants (Moore et al., 1987; Roberts and Lipman, 2006; Levison and Levison, 2009; van Maarseveen et al., 2016). Nevertheless, to date none of the existing guidelines and recommendations regarding the dose rationale for gentamicin have been developed taking these factors into account in a strictly quantitative manner. Such an empiricism in the dose rationale cannot be overlooked. Clearly, in some cases, the use of linear dosing algorithms, such as doses in mg/kg body weight may result in sub-optimal or undesirable drug levels across the target population (Hansen et al., 2003; Neef et al., 2006; Rocha et al., 2007; Zakova et al., 2014).

We acknowledge that very few studies have evaluated clinical response taking into account pharmacokinetic variability and so far no data on drug levels have been collected after administration of gentamicin in a resource-limited setting (Dersch-Mills et al., 2012). However, we believe that extrapolation of the pharmacokinetic parameters from a hospital setting to out-patient protocols, as described in the current investigation can be performed with sufficient precision to assess the impact of covariates on drug disposition, irrespective of the treatment setting (Thomson et al., 2003; Roberts and Lipman, 2006; Fuchs et al., 2014). As highlighted in previous paragraphs, we also recognize that assumptions need to be made about the role of other intrinsic and extrinsic sources of variability (e.g., compliance, disease severity, age of onset), which were not included in our analysis (Garcia et al., 2006; Thingvoll et al., 2008; Marsot et al., 2012). For example, information about serum creatinine was not available in the AFRINEST and SATT trials data sets, nor was it included as a covariate on gentamicin elimination parameters in the model developed by Fuchs et al. However, changes in clearance due to renal maturation are captured by the maturation function, expressed in terms of the effect of gestational and postnatal age. Obviously, the maturation function cannot explain differences associated with organ impairment, which may exist due to the presence of co-morbidities or complications due to worsening of sepsis. In such cases, doses would need to be adjusted based on the same principles used for renal impairment. We have also had to use predefined correlations between body weight, gestational and post-menstrual age in preterm and term newborns and infants, which may not fully replicate the variation in a real-world setting, where the correlation between body weight, gestational and post-menstrual age may be further affected by other extrinsic factors, such as malnutrition and disease severity (e.g., diarrhea). We anticipate that these limitations should not alter the conclusions drawn from the current analysis. Furthermore, we should highlight that the use of single cut-off value for the selection of the doses for each weight band created a rather stringent criterion for treatment performance, as microbiological susceptibility data would not be available in a setting where referral is not possible. In reality, ranges have been used for C_max_ (e.g., 8–12 mg/ml or 6–15 mg/ml) along with varying dosing intervals to ensure both peak and trough target levels are achieved in most neonatal patients (Touw et al., 2009; Martínková et al., 2010; Yu et al., 2020). Nevertheless, we recommend the use of sparse sampling schemes in prospective clinical trials to confirm the predicted pharmacokinetic profiles and ensure the effective implementation of the proposed dosing regimen for the treatment of sepsis in newborns and infants.

The reader should be aware that the recent WHO guidelines have been developed taking into account the existing evidence, from clinical practice and randomised clinical trials in neonates (0–28 days old) and young infants (0–59 days old) with severe bacterial infection in resource-limited settings, where families do not accept or cannot access referral care (World Health Organization, 2015). Whereas the goal of such guidelines is to provide clinical guidance on the simplest antibiotic regimens that are both safe and effective for outpatient treatment of clinically severe infections in children 0–59 days old, it appears that an opportunity has been missed to ensure that recommendations are supported by a dose rationale based on an integrated analysis of pharmacokinetics and PKPD principles.

In summary, our findings are promising in that a simpler dosing regimen can be implemented in community-based settings. Intramuscular gentamicin can be used as a fixed dose according to a weight-banded regimen. The proposed regimen for neonates and young infants with sepsis aged 0–59 days differs from current guidelines in that it takes into account the effect of body weight, gestational age and post-natal age as determinants of the variability in the pharmacokinetics of gentamicin. A dose rationale that accounts for the role of influential factors on drug disposition represents a major advancement in the treatment of possible serious bacterial infections in resource-limited settings.

## Data Availability

The simulated data sets generated for this study are available on request to the corresponding author.
