# Supplementary material for: Simplified Dosing Regimens for Gentamicin in Neonatal Sepsis
Source: Front Pharmacol. 2021 Mar 8;12:624662. doi: 10.3389/fphar.2021.624662 (PMC7982486; doi:10.3389/fphar.2021.624662)
Supplement: Supplementary file 1 [file datasheet1.pdf]

Supplemental material

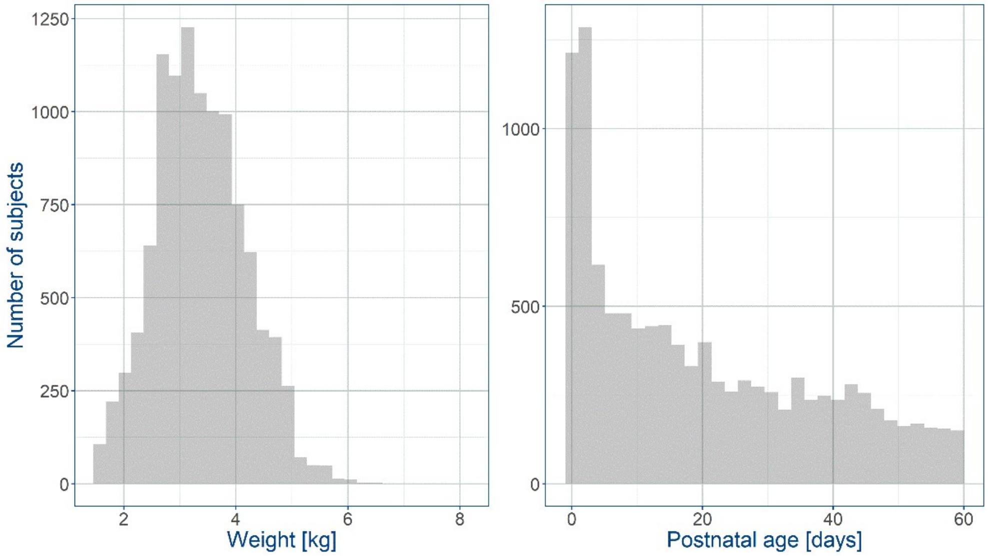

Figure S1. Histograms show weight and postnatal age distributions for the AFRINEST and SATT trial populations. Individual postnatal age and body weight data were used in conjunction with predicted gestational age to simulate gentamicin pharmacokinetic profiles in neonates and young infants with sepsis.

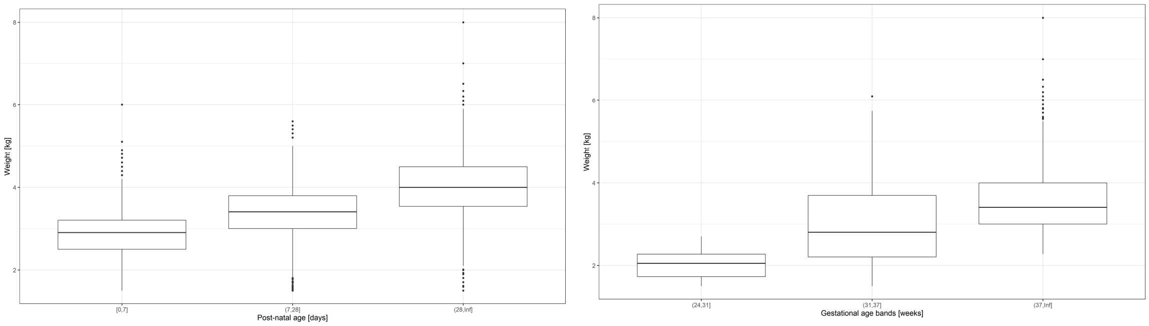

Figure S2. Whisker box plots showing the median, quartiles and 90% confidence intervals of the correlations between body weight and post-natal age (left panel) and imputed gestational age (right panel) based on the approach proposed by Sumpter and Holford (2011) which assumes a correlation between actual body weight, gestational and post-natal age. Individual gestational age values were inferred from the observed individual body weight and median gestational age for the patient’s body weight.

Table S1. Selected publications in which gentamicin pharmacokinetics was characterized using compartmental methods and nonlinear mixed effects modelling. The highlighted publication by Fuchs *et al.* was used for the implementation of the simulation scenarios.

Publication	No. patients / No. patients with sepsis	Sepsis	Dosing regimen	PNA (days)	GA (weeks)	BW range (kg)	Structural model	Covariates	Method for model validation
Sherwin CM *et al.* Biopharm Drug Dispos. 2009; 30(5):276-80.	116/15	YES	not reported	4.4 ± 8.4	34.7 ± 5.43	2.4 ± 1.2	1 CPT	Body weight on CL and V<br>Sepsis on V	Bootstrap
Lingvall M *et al.* Br J Clin Pharmacol. 2005; 59(1):54-61.	277/15	YES	2.5-4mg/kg i.v.	0-21	28-41	1-4.2	1 CPT	Body weight and age on CL and V<br>Sepsis on V	Basic GOF plots
Thomson AH *et al.* Br J Clin Pharmacol. 2003; 56(1):25-31.	107/107	YES	8mg/kg i.m	0-99	NA	1.2-6.7	1 CPT	NA	NA
Fuchs A *et al.* Br J Clin Pharmacol. 2014; 78(5):1090-101.	1508/0	NO	3-4mg/kg i.v.	0-94	24-42	0.4-5.5	2 CPT	Body weight and age on CL and V	Visual predictive checks, internal and external validation
Nielsen EI *et al.* Clin Pharmacokinet. 2009; 48(4):253-63.	61/0	NO	3-4mg/kg i.v.	0-45	24-41	0.49-5.1	3 CPT	Body weight and age on CL	Visual predictive checks

Table S2. Parameter estimation of final pharmacokinetic model in Fuchs et al.

Parameters	Estimates	SE (%)
**CL** $(\boldsymbol{L \cdot h^{-1}})$	0.089	1*
**θ_{CLBW}**	0.75	N/A
**θ_{CLGA}**	1.87	3*
**θ_{CLPNA}**	0.054	6*
**θ_{CLDOPA}**	-0.120	22*
**V_c** $(\boldsymbol{L})$	0.908	2*
**θ_{VcBW}**	1	N/A
**θ_{VcGA}**	-0.922	8*
**Q** $(\boldsymbol{L \cdot h^{-1}})$	0.157	7*
**θ_{QBW}**	0.75	N/A
**V_p** $(\boldsymbol{L})$	0.56	4*
**θ_{VpBW}**	1	N/A
**BSV CL (%)**	28	3†
**BSV V_c (%)**	18	1†
**Correlation CL-V_c (%)**	87	3
**Additive residual error** $(\boldsymbol{mg \cdot L^{-1}})$	0.1	24*
**Proportional residual error (%)**	18	1*

*Standard errors of the estimates (SE), defined as SE/estimate and expressed as percentages. †Standard errors of the coefficient of variation, taken as SE / (Estimates × 2) and expressed as percentage. BSV, between subject variability; BW, body weight; CL, clearance; DOPA, dopamine; GA, gestational age; PNA, post-natal age; Q, inter-compartmental clearance; V_c, central volume of distribution; V_p, peripheral volume of distribution. *[Adapted from Fuchs et al., 2014 with permission]*.

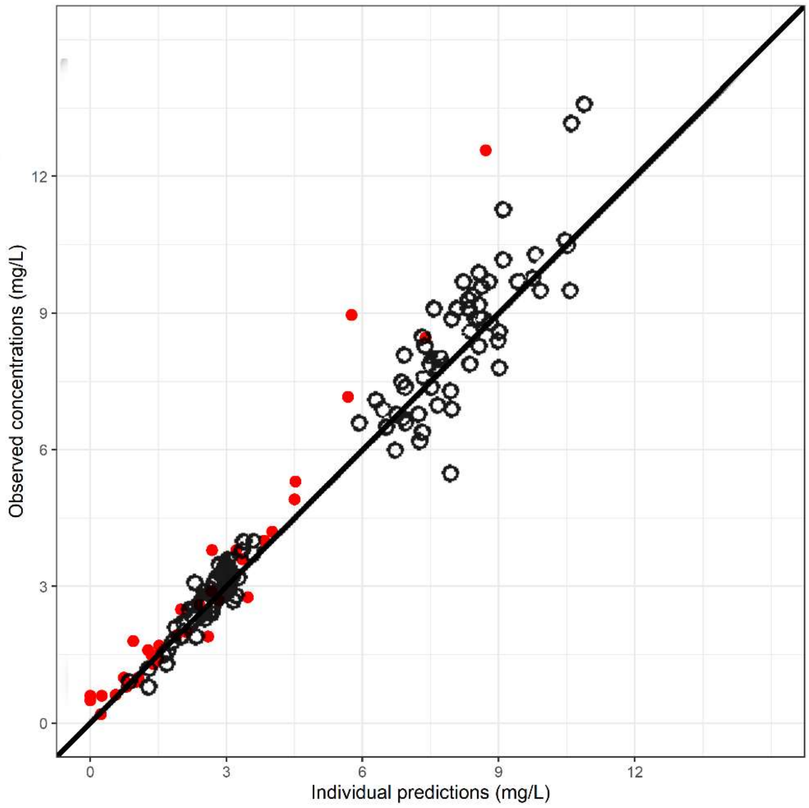

Figure S3. Goodness-of-fit plot showing the model individual predictions vs. observations. Black empty circles represent the results of the external validation by Fuchs *et al.*, 2014. Red circles describe the data from 28 subjects included in the secondary external validation. Black line depicts the identity line. *[Adapted from Fuchs et al., 2014 with permission]*

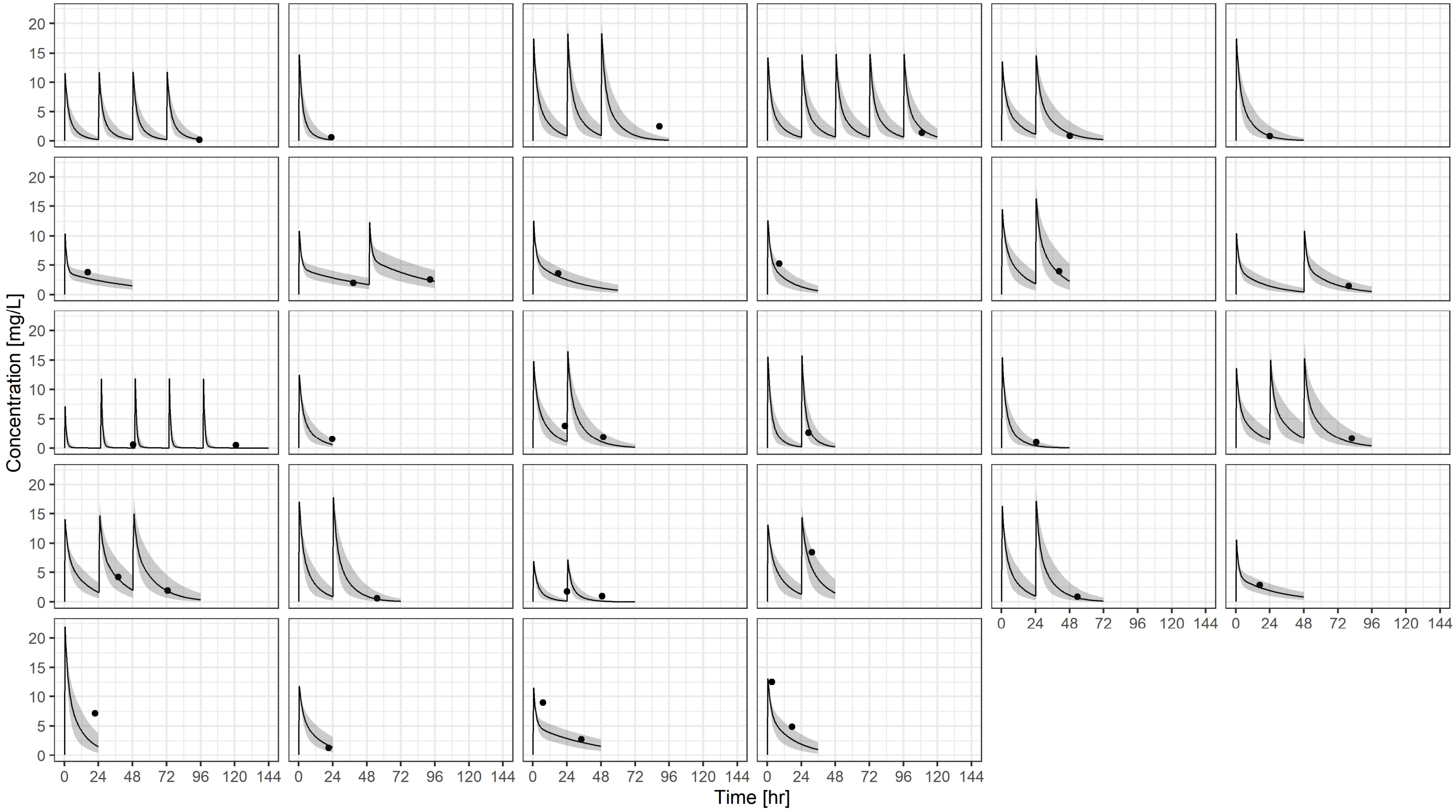

Figure S4. Individual visual predictive checks for the subjects included in the external validation data set. Circles represent the observed gentamicin concentration. Solid line depicts the predicted median profile; shaded area represents the 90% prediction intervals.

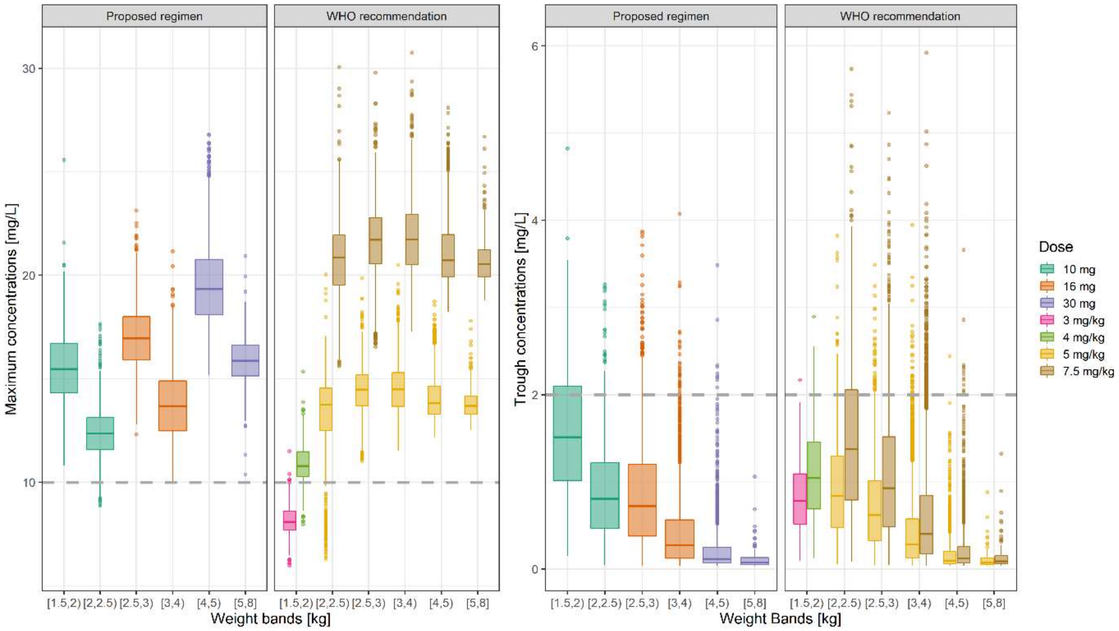

Figure S5. Assessment of the suitability of the 2015 WHO recommendation and proposed simplified regimen based on target exposure achieved within narrower weight bands. Panels show the predicted gentamicin peak (left) and trough (right) concentrations in sepsis patients aged between 0 – 59 days.

Hinges represent 25th and 75th percentiles (respectively, Q1 and Q3), whiskers represent Q1 –1.5IQR and Q3 + 1.5IQR, respectively, where IQR is the interquartile range. All the subjects outside this range are represented by the dots (N= 9994). Dashed lines represent the threshold value for peak (10 mg/L) and trough (2 mg/L) concentrations, respectively. These results suggest that the regimen currently recommended by the WHO for newborn infants <2 kg results in a considerable number of patients below the target threshold for peak concentrations.
